# Meandering journey towards routine trial adaptation: survey results on barriers to use of adaptive designs in confirmatory trials

**DOI:** 10.1186/1745-6215-16-S2-O20

**Published:** 2015-11-16

**Authors:** Munyaradzi Dimairo, Steven Julious, Susan Todd, Jonathan Nicholl

**Affiliations:** 1 University of Sheffield, School of Health and Related Research, Sheffield, UK; 2 University of Reading, Department of Mathematics and Statistics, Reading, UK

## Background

Adaptive designs (ADs) are innovative alternative trial designs with the potential to improve efficiency in clinical trials research when used appropriately. Despite this, their implementation has been slow to gain traction. This study explored barriers, concerns and potential facilitators to appropriate use of ADs in confirmatory trials.

## Methods

Based upon themes drawn from in-depth interviews of key research stakeholders, we undertook cross-sectional, online surveys (November 2014 to January 2015) of; CTUs, public funders and industry in the UK. Observed response rates were: 55% (30/55) CTUs, 68% (17/25) industry, and 41% (86/212) public funders. We employed Rating Scale Modelling [5] to rank the perceived importance of barriers and concerns for prioritisation.

## Results

Most important perceived barriers to adoption in the public sector included: the lack of bridge funding to support design developmental work of time consuming ADs, dearth of practical knowledge and experience, lack of applied training to facilitate practical learning, preference for traditional designs, time constraints to support planning, insufficient access to case studies, difficulties in marketing ADs to key research stakeholders and associated practical complexities.

Respondents' perceptions of ADs were consistent across industry. The exceptions, including bridge funding support, inadequate data management infrastructure and regulatory fear, were probably reflections of differential organisational structures, experience, and type of research interventions.

## Conclusions

There are multifaceted barriers and concerns to the appropriate use of ADs most of which are intertwined with lack of practical knowledge and experience. Learning from “pacesetters” through applied training and accessible publication of ADs undertaken coupled with adequate transparent reporting is paramount. There is an urgent need for a practical toolkit of design specific questions researchers need to ask themselves when considering planning particular ADs and a consensus guidance document on ADs tailored for publicly funded confirmatory trials.

